# ICAM-1-Based Rabies Virus Vaccine Shows Increased Infection and Activation of Primary Murine B Cells *In Vitro* and Enhanced Antibody Titers *In-Vivo*

**DOI:** 10.1371/journal.pone.0087098

**Published:** 2014-01-29

**Authors:** James E. Norton, Andrew G. Lytle, Shixue Shen, Evgeni P. Tzvetkov, Corin L. Dorfmeier, James P. McGettigan

**Affiliations:** 1 Department of Microbiology and Immunology, Jefferson Medical College, Thomas Jefferson University, Philadelphia, Pennsylvania, United States of America; 2 Jefferson Vaccine Center, Jefferson Medical College, Thomas Jefferson University, Philadelphia, Pennsylvania, United States of America; Thomas Jefferson University, United States of America

## Abstract

We have previously shown that live-attenuated rabies virus (RABV)-based vaccines infect and directly activate murine and human primary B cells *in-vitro*, which we propose can be exploited to help develop a single-dose RABV-based vaccine. Here we report on a novel approach to utilize the binding of Intracellular Adhesion Molecule-1 (ICAM-1) to its binding partner, Lymphocyte Function-associated Antigen-1 (LFA-1), on B cells to enhance B cell activation and RABV-specific antibody responses. We used a reverse genetics approach to clone, recover, and characterize a live-attenuated recombinant RABV-based vaccine expressing the murine *Icam1* gene (rRABV-mICAM-1). We show that the murine ICAM-1 gene product is incorporated into virus particles, potentially exposing ICAM-1 to extracellular binding partners. While rRABV-mICAM-1 showed 10-100-fold decrease in viral titers on baby hamster kidney cells compared to the parental virus (rRABV), rRABV-mICAM-1 infected and activated primary murine B cells *in-vitro* more efficiently than rRABV, as indicated by significant upregulation of CD69, CD40, and MHCII on the surface of infected B cells. ICAM-1 expression on the virus surface was responsible for enhanced B cell infection since pre-treating rRABV-mICAM-1 with a neutralizing anti-ICAM-1 antibody reduced B cell infection to levels observed with rRABV alone. Furthermore, 100-fold less rRABV-mICAM-1 was needed to induce antibody titers in immunized mice equivalent to antibody titers observed in rRABV-immunized mice. Of note, only 10^3^ focus forming units (ffu)/mouse of rRABV-mICAM-1 was needed to induce significant anti-RABV antibody titers as early as five days post-immunization. As both speed and potency of antibody responses are important in controlling human RABV infection in a post-exposure setting, these data show that expression of *Icam1* from the RABV genome, which is then incorporated into the virus particle, is a promising strategy for the development of a single-dose RABV vaccine that requires only a minimum of virus.

## Introduction

Rabies virus (RABV) causes a deadly zoonotic infection that targets and causes dysfunction within the central nervous system (CNS) of infected hosts. Upon manifestation of symptoms, rabies is nearly always fatal [1]. It is estimated that RABV is responsible for 55,000 human deaths per year worldwide, though this number may be much larger [2]. Most of the disease burden is located in the developing nations of Asia and Africa, where it is estimated that 3.3 billion people live at risk of RABV infection [2]. Of those infected, 40% are under 15-years-of-age [2]. Over 15 million people receive post-exposure prophylaxis (PEP) after exposure to a potentially infected animal [2]. If administered in a timely and appropriate manner, current PEP is nearly 100% successful in preventing human RABV infection. This, together with routine vaccination of domestic animals, has resulted in a dramatic reduction of human RABV infections in developed countries over the last 50–60 years [3].

Current, standard PEP for previously unvaccinated, immunocompetent individuals includes prompt wound cleaning and the administration of four to five doses of inactivated vaccine, and in the case of severe exposure, one dose of rabies immune globulin (RIG) [2], [4]. The efficacy of rabies PEP in developing countries where rabies is highly endemic is hindered by high costs and a lack of compliance, which emphasize the need for a single-dose RABV-based vaccine to combat this global public health threat [reviewed in [5], [6]. However, it does not appear that this single-dose vaccine will be based on currently available inactivated vaccines. A recent study by *Strady et. al*. showed that a minimum of three doses of the current inactivated RABV vaccine are required to reduce the percentage of non- or poor-responders [less than 0.5 international unit (IU)/ml] to just 3% in a pre-exposure setting [7]. In contrast, live attenuated virus vaccines tend to be much more immunogenic than their inactivated counterparts as shown in non-human primates immunized with a replication-deficient RABV-based vaccine compared to the commercially available Human Diploid Cell Vaccine (HDCV) [8].

To help delineate factors that contribute to the efficacy of live attenuated RABV-based vaccines in the context of B cell activation, immunity and protection, we recently showed that live, highly attenuated RABV-based vaccine vectors induce T cell-independent and extrafollicular T cell-dependent B cell responses that provide protection against pathogenic RABV challenge [9]. Importantly, vaccine-induced IgM helps to prevent the spread of a pathogenic RABV strain to the CNS and thereby contributing to protection within days of immunization [10]. We also showed that RABV-based vaccines efficiently infect naïve primary murine and human B cells *ex-vivo*, resulting in the significant upregulation of early markers of B cell activation and antigen presentation, including CD69, MHCII, and CD40 in murine B cells or HLA-DR and CD40 in human B cells compared to cells treated with an inactivated RABV-based vaccine [11]. Primary B cells infected with a live attenuated RABV expressing ovalbumin directly prime and stimulate naïve CD4^+^ OT-II T cells to proliferate and to secrete IL-2, demonstrating an important functional consequence of B cell infection and activation by live RABV-based vaccine vectors [11]. We propose that this direct B cell stimulation by live RABV-based vaccines is a potential mechanism underlying their induction of early, protective B cell responses, and that live RABV-based vaccines designed to infect and activate B cells represent a promising strategy to develop a single-dose post-exposure rabies vaccine where the generation of early protective antibody titers is critical.

To this end, in this report, we investigated the utility of incorporating ICAM-1 into a RABV-based vaccine to target B cells and thereby improve B cell infection, activation and anti-RABV humoral immunity. ICAM-1 (CD54) is a transmembrane protein of the immunoglobulin (Ig) superfamily of proteins [12] whose binding partners include LFA-1 (CD18/CD11a) and macrophage-1 antigen [(MAC-1); (CD18/CD11b)], both of which are integrin family members [13], as well as the clotting protein fibrinogen. ICAM-1 is present on various cell lineages including leukocytes, dendritic cells (DCs), follicular dendritic cells (FDCs), and endothelial cells [14]. LFA-1 is present on leukocytes, including B cells, DCs, and natural killer (NK) cells [15]. Traditionally, ICAM-1 has been known to participate in cell-cell adhesion, cell migration, and extravasation of immune cells into sites of inflammation. However, the function of ICAM-1 in immunity is much more complex. ICAM-1 has been implicated as an additional costimulatory molecule involved in T cell stimulation [16], [17]. Cooperative signaling through ICAM-1 interaction may be required for optimum response of CD4 T cells to MHCII-presented peptide antigen [18].

In addition to the expression and function of ICAM-1 on CD4 T cells to stabilize interactions with cells bearing MHCII-presented antigens, ICAM-1 may play important roles in viral immunology. Specifically, ICAM-1 is naturally incorporated into HIV-1 particles and enhances infection of LFA-1-expressing cells by stabilizing virus:cell interactions and supporting viral uptake [19]–[22], suggesting the incorporation of ICAM-1 into a virus that can target B cells, such as attenuated RABV strains [11], may help to enhance B cell infection and activation. In addition, *Carrasco et. al.* showed that upon BCR engagement, LFA-1 on the surface of B cells is recruited to the BCR synapse, where lipid bilayer-anchored ICAM-1 can bind to LFA-1 and lower the antigen threshold required for B cell activation [23]. This suggests vaccine strategies that exploit ICAM-1/LFA-1 binding interactions on B cells may promote effective B cell immunity with a minimal vaccine dose. Finally, *Denning et. al*. showed that B cells with higher levels of surface ICAM-1 expression, which might be the case for B cells infected with a recombinant RABV-based vaccine expressing ICAM-1, stimulate T cells to a greater magnitude than do B cells expressing low levels of surface ICAM-1 [24]. Together, an ICAM-1-based RABV vaccine holds the potential to influence B cells responses directly by aiding in B cell infection, B cell activation and B:T interactions, resulting in enhanced anti-RABV antibody responses.

In this report, we exploited the potential immune enhancing features of ICAM-1 expression in the context of RABV vaccination. We constructed, grew and characterized a live-attenuated rRABV-based vaccine expressing the murine *Icam1* gene (rRABV-mICAM-1). When assessed *in-vitro*, rRABV-mICAM-1 showed greater infection and activation of naive primary mouse B cells compared to a parental rRABV-based vaccine strain. In addition, mice primed intramuscularly with one low-dose of rRABV-mICAM-1 showed higher, more rapid antibody responses *in-vivo* as early as five days post-immunization compared to mice primed with the same dose of rRABV. Taken together, these data indicate that rRABV-mICAM-1 is capable of significant infection and activation of B cells and is capable of inducing high, early antibody titers using only one, low-dose inoculation. As such, expression of *Icam1* from the RABV genome is a promising strategy for the development of a single-dose RABV vaccine.

## Materials and Methods

### Ethics statement

All animal work was reviewed and approved by the Institutional Animal Care and Use Committee (IACUC) of Jefferson Medical College, Thomas Jefferson University. Work was completed in accordance with international standards [Association for Assessment and Accreditation of Laboratory Animal Care (AAALAC)] and in compliance with Public Health Service Policy on Humane Care and Use of Laboratory Animals, The Guide for the Care and Use of Laboratory Animals of the National Institutes of Health (NIH).

### Recombinant RABV-based vaccine construction and recovery

rRABV is a recombinant RABV-based vaccine vector and is a molecular clone of the SAD-B19 vaccine strain of RABV [25], [26]. To construct rRABV expressing ICAM-1, the *Icam1* gene of *Mus musculus* encoded in pBlueScript SK^+^ (ATCC) was amplified by polymerase chain reaction (PCR) with VENT polymerase [New England Biolabs (NEB)] using forward primer JPM24 (5′ – TTTCGTACGATTATGGCTTCAACCCGTGCCAAG – 3′) (*BsiWi* underlined) and reverse primer JPM25 (5′ – AAATCTAGATCAGGGAGGTGGGGCTTG – 3′) (*XbaI* underlined). The PCR product was digested with *BsiW1* (NEB) and *Xba1* (NEB) and ligated into the plasmid prRABV that had previously been digested with *BsiW1* and *Nhe1* (NEB), resulting in a plasmid named prRABV-ICAM-1. Infectious virus was recovered following standard methods to recover infectious RABV-based vaccines from cDNA [25], [27]. Virus was grown on baby hamster kidney cells and then purified by ultracentrifugation over a 20% sucrose cushion to remove soluble ICAM-1 (sICAM-1) contained in the supernatants of the produced virus.

### Vaccine characterization

#### Western Immunoblotting and Silver Stain

BSR cells (a derivative of a baby hamster kidney cell line) were infected with rRABV or rRABV-mICAM-1 at a multiplicity of infection (MOI) of 2 focus-forming units (ffu). Uninfected cells served as negative controls. Cells were incubated at 37°C with 5% CO_2_ overnight. Cells were lysed with RIPA buffer (Sigma-Aldrich) and cellular debris pelleted out. Alternatively, sucrose-purified rRABV or rRABV-mICAM-1 were lysed with RIPA buffer. Proteins were separated via 10% SDS-PAGE and transferred to a polyvinylidene membrane. The membrane was blocked with 5% nonfat milk powder in 1X PBS for 1 hour at room temperature. The membrane was incubated with 0.1 µg/ml of polyclonal goat IgG murine ICAM-1 (R&D Systems, Inc.) in Western blot (WB) wash buffer (PBS, 0.05% Tween 20) at 4°C overnight. The membrane was washed three times with WB wash buffer and incubated with secondary antibody (donkey anti-goat IgG horseradish peroxidase; Jackson ImmunoResearch Laboratories) at 1∶50,000 in WB wash buffer for 1 hour at room temperature. The membrane was washed three times with WB wash buffer and twice with 1X PBS prior to performing a chemiluminescence assay (Thermo Fisher Scientific, Inc.) as instructed by the manufacturer. To help confirm incorporation of ICAM-1 into the RABV particle, virus particles were first purified over a 20% sucrose cushion and the purified virus was loaded onto a sucrose step gradient (20% to 40% in 5% increments) and then centrifuged for 2 hours at >48K g. Virus particles were isolated from the single band located at the 35–40% interface. Proteins were separated via 10% SDS-PAGE and then visualized by silver staining as described by the manufacturer (Pierce). The gel was scanned and then converted to grayscale for publication.

#### One- and Multi-Step Growth Curves

Growth kinetics of rRABV and rRABV-mICAM-1 were determined in parallel as previously described [28]. Briefly, BSR cells were seeded at 5.0×10^5^cells/well in 6-well plates (Corning, Inc.) in Dulbecco's Modification of Eagle's Media (DMEM) containing 5% heat-inactivated FBS/1% Penicillin/Streptomycin and infected 24 hours later with rRABV or rRABV-mICAM-1 at a MOI of 5 or 0.01 for one-step or multi-cycle growth curves, respectively. After a two hour incubation, cells were extensively washed with 1X PBS, and supplemented with DMEM. Tissue culture supernatants (100 µl) were harvested at the indicated time points. Titers of infectious supernatants were determined using BSR cells in duplicate.

### *In-vitro* infection, activation and flow cytometry analysis of primary murine splenocyte cultures

#### In-vitro infection

Single cell suspensions (10^6^ cells/ml) from spleens of naïve female C57BL/6 mice aged 6–8 weeks were cultured in splenocyte medium (RPMI 1640 containing 10% FBS, 50 mM beta-mercaptoethanol, 100 IU/mL Penicillin/Streptomycin, and 100 mM HEPES). Cells were infected with rRABV, rRABV-mICAM-1, or media alone (mock-infected) at a MOI of 5 and incubated at 37°C with 5% CO_2_ for two days in the absence of additional mitogens to maintain the B cells and accessory splenocytes in a resting state similar to that in which they would exist *in-vivo* at the time of initial immunization [11].

#### Immunostaining and Flow Cytometry

Infected or mock-infected cells were harvested two days post-infection (p.i.). Cells were washed with FACS buffer (PBS containing 2% heat-inactivated FBS) and stained with fluorophore-conjugated antibodies. Cellular surface markers stained included CD40, CD45R (B220), CD69, and MHCII (all from eBioscience). Cells were washed twice with FACS buffer and fixed in 2% paraformaldehyde at 4°C for 30 min. Cells were washed twice with FACS buffer and once with PermWash permeabilization buffer (BD Biosciences) for intracellular staining. Cells were incubated in PermWash containing FITC-conjugated RABV nucleoprotein (N)-specific antibody (Fujirebio Diagnostics, Inc.) and phycoerythrin-conjugated anti-murine ICAM-1 antibody (R&D Systems, Inc.) at 4°C for 15 min. Cells were washed twice with FACS buffer and resuspended in FACS buffer prior to flow cytometry analysis. Fluorescently-labeled cells were analyzed by FACScan [(BD LSRII; BD Biosciences); (Kimmel Cancer Center, Jefferson Flow Cytometry Facility, Jefferson Medical College, Thomas Jefferson University)] and FlowJo software (TreeStar, Inc.) [11], [29]. To determine the effects of blocking ICAM-1 function on the surface of virus particles, sucrose-purified rRABV-mICAM-1 virions were incubated with anti-mICAM-1 antibody (5 µg/ml; R&D Systems, Inc.) for 2 hours at 37°C with 5% CO_2_. Single cell suspensions of murine splenocytes were prepared and treated with virus:antibody mix for two days and then stained as described above for RABV N, B220 and MHCII. To compare two groups of data, we used an unpaired, two-tailed Student's t test. (* p<0.05; **, p<0.01; ***, p<0.001) (N = 3 completed in duplicate per group).

### *In-vivo* immunogenicity studies

#### Immunization

Groups of 5 naïve C57BL/6 female mice aged 6–8 weeks (National Cancer Institute, National Institutes of Health, Bethesda, MD) were immunized intramuscularly (i.m.) in the hind legs with 10^3^ ffu of rRABV or rRABV-mICAM-1. Additional groups of mice were immunized as just described except using a higher vaccine dose (10^5^ ffu). PBS-immunized mice served as negative controls.

#### Sample collection and immune assays

Blood was collected from all mice by retro-orbital eye bleed on various days post-immunization. Sera were analyzed for anti-RABV G total IgG and IgM antibody titers by ELISA as previously described [30], [31]. Statistical difference in antibody titers by ELISA between two groups of data was determined using an unpaired, two-tailed t test and data is presented at the mean ± SEM. *p<0.05, **p = 0.01−0.001, ***p≤0.001. (N = 5 mice per group). Virus-neutralizing antibody titers were performed using the Modified Rapid Fluorescent Focus Inhibition Test (RFFIT) as previously described [8], [32]. Neutralization titers were normalized to IU/ml using the World Health Organization standard human anti-rabies immunoglobulin reference.

## Results

### Construction, Recovery and Characterization of a rRABV-based Vaccine Vector Expressing Murine *Icam1* Gene (rRABV-mICAM-1)

**Figure 1:**
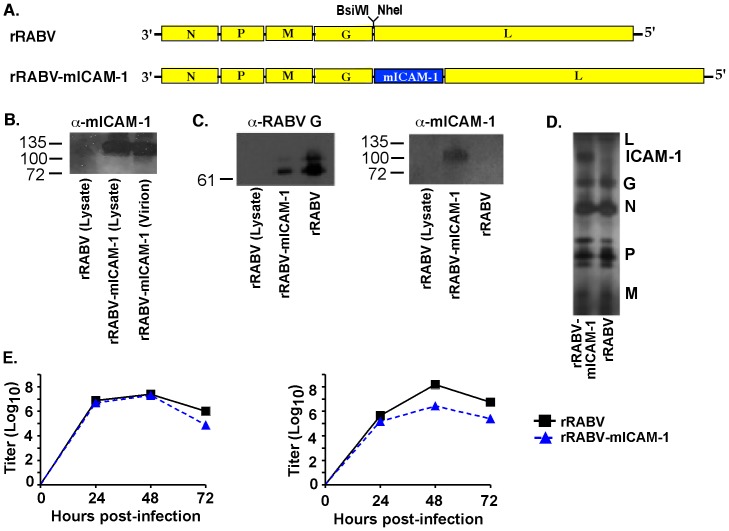
Construction, recovery and characterization of a recombinant RABV-based vaccine expressing the murine *Icam1* gene (rRABV-mICAM-1). **A)** At the top is a parental recombinant RABV-based vaccine containing an additional transcription stop-start signal flanked by two unique restriction sites between the G and the L genes. rRABV, which is a molecular clone of the SAD-B19 vaccine strain of RABV, was the target to introduce the gene encoding murine ICAM-1. **B)**. BSR cells, which are not expected to express ICAM-1 or its binding partner LFA-1, were infected with rRABV or rRABV-mICAM-1 and lysed 48 hours later. Proteins were separated by SDS-PAGE and subjected to Western blotting with antibodies specific for ICAM-1. A protein of the expected size for ICAM-1 was detected from lysates of rRABV-mICAM-1-infected but not from rRABV-infected BSR cells. In parallel, sucrose-purified and concentrated virus was analyzed by western blot analysis and a protein of the expected size for ICAM-1 was detected in the virion particle. **C)** Sucrose-purified rRABV-mICAM-1 was re-purified using 5% sucrose step gradient and the single band was collected and analyzed by Western blot analysis to show ICAM-1 protein migrated within the sucrose gradient at the same density as rRABV. RABV and lysates from uninfected BSR cells served as controls for the Western blot analyses. **D)** Silver staining of separated proteins from sucrose step gradient-purified particles shows a protein at the expected size of ICAM-1 and that the incorporation of ICAM-1 into the virus particles does not affect viral protein compositions. **E)** BSR cells were infected with rRABV or rRABV-mICAM-1 at a MOI of 5 (one step growth; left) or .01 (multi-cycle growth curve; right). Aliquots of tissue culture supernatants were collected, and viral titers were determined in duplicate.

We previously showed that live RABV-based vaccines are highly immunogenic in mice and non-human primates [8], [31], [32]. We also showed that live RABV-based vaccines infect naïve primary murine and human B cells, resulting in B cell activation and the ability of B cells to prime naïve T cells to proliferate and secrete IL-2 [11]. To determine whether B cell infection and activation could be enhanced by expressing murine ICAM-1 from a rRABV-based vaccine *in-vitro*, and whether enhanced B cell infection and activation leads to improved anti-RABV antibody responses *in-vivo*, we constructed and recovered a recombinant, attenuated vaccine strain of RABV expressing the murine *Icam1* gene (rRABV-mICAM-1, Figure 1A). Western blot analysis of lysed BSR cells, which do not express endogenous ICAM-1 or its binding partner LFA-1, infected with rRABV or rRABV-mICAM-1 confirmed expression of the *Icam1* gene from rRABV-mICAM-1 but not rRABV or uninfected cells (Figure 1B). Western blot analysis of sucrose-purified and concentrated virus showed that the ICAM-1 protein was incorporated into the rRABV-mICAM-1 viral particle (Figure 1B). To help confirm that ICAM-1 was incorporated into the RABV particle, sucrose-purified rRABV or rRABV-mICAM-1 were isolated on a 5% step sucrose gradient and the single band collected. Western blot analysis detected ICAM-1 from the same density within the gradient as RABV (Figure 1C), indicating that ICAM-1 migrates with RABV in the sucrose gradient. To determine whether incorporation of ICAM-1 into the virus affects the content of viral proteins within the particle, we completed silver staining of the step gradient-purified particles. As shown in Figure 1D, similar levels of each viral protein were detected in rRABV or rRABV-mICAM-1 particles indicating incorporation of ICAM-1 does not affect the composition of viral proteins. While it was previously determined that the RABV G cytoplasmic domain is required for incorporation of a foreign protein into the RABV particle [33], this requirement is not absolute, as demonstrated here in the case of ICAM-1 expression and elsewhere [34]. One-step and multi-cycle growth curves were prepared to measure replication rates of rRABV-mICAM-1 compared to rRABV in BSR cells. Viral titers of supernatants harvested from cells infected with a high multiplicity of infection (MOI = 5; one-step growth curves) show that rRABV-mICAM-1 produces progeny virus at similar levels and with similar kinetics as does rRABV (Figure 1E, left panel). However, viral titers of supernatants harvested from cells infected with a low multiplicity of infection (MOI) (MOI = 0.01; multi-cycle growth curves) show that rRABV-mICAM-1 is slightly spread-deficient compared to rRABV, as indicated by 10–100-fold lower viral titers (Figure 1E, right panel). Since BSR cells are not expected to express a ligand for mICAM-1, differences in growth kinetics most likely arise from the insertion of a foreign gene and not due to ICAM-1 function. Together, an infectious live RABV-based vaccine vector that expresses the murine ICAM-1 protein from the viral genome, which is incorporated into viral particles, was cloned and recovered and shown to be somewhat deficient in its ability to spread *in-vitro*.

### rRABV-mICAM-1 Infects Primary Murine B Cells *In-vitro* More Effectively Than Does rRABV

**Figure 2:**
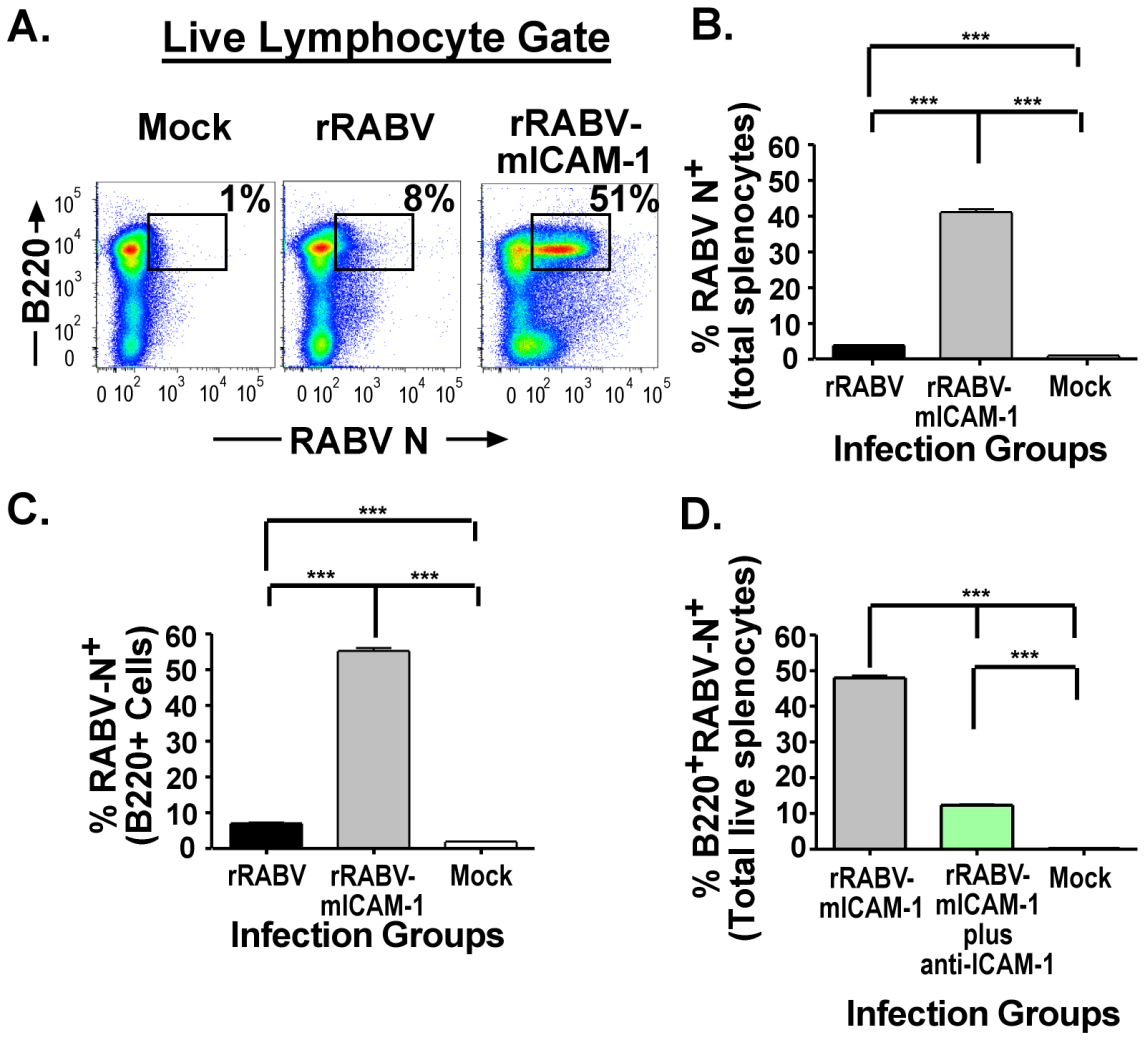
rRABV-mICAM-1 infects primary murine splenocytes and B cells more efficiently than the parental virus, rRABV. Naïve primary murine splenocytes were infected at an MOI of 5 with rRABV or rRABV-mICAM-1, or treated with media alone (mock-infected) for two days *in-vitro*. No additional mitogens were added to the culture to maintain the B cells and accessory splenocytes in a resting state similar to that in which they would exist *in-vivo* at the time of initial immunization. **A)** Representative gating strategy of total live splenocytes stained for intracellular RABV N as a marker for infection and for cell-surface expression of B220 as a maker for B cells. The percentage in the upper right quadrant is a representative example of the percentage of B220^+^ B cells infected with the rRABV-based vaccine. **B)** Percent RABV N^+^ cells in the total live lymphocyte gate. **C)** Percent RABV N^+^ cells in the B220^+^ cell population. **D)** Pretreatment of sucrose-purified rRABV-mICAM-1 with a neutralizing anti-ICAM-1 antibody significantly reduced infection of B cells in culture. To compare two groups of data, we used an unpaired, two-tailed Student's t test. (* p<0.05; **, p<0.01; ***, p<0.001)

As noted previously, HIV-1 naturally incorporates ICAM-1 into HIV-1 particles, which enhances infectivity of LFA-1-expressing target cells [19]–[21]. Based on this finding, and our previous findings that live attenuated RABV vaccine strains infect and activate primary murine and human B cells *in-vitro*, we hypothesized that a recombinant RABV vaccine vector that expresses and incorporates ICAM-1 into the viral particle would promote infection of B cells, resulting in enhanced B cell activation *in-vitro*. Naïve primary murine splenocytes were infected at an MOI of 5 with rRABV or rRABV-mICAM-1, or treated with medium alone (mock infected) for two days *in-vitro*. No additional mitogens were added to the culture to avoid expressing activation molecules that could enhance sensitivity to RABV infection and activation [11]. Figure 2A shows a representative gating strategy of total live splenocytes stained for RABV N as a marker for infection and for cell-surface expression of B220 as a maker for B cells [9], [29]. Consistent with our previous data showing that live rRABV-based vaccines infect primary murine splenocytes [11], approximately 5% of the total splenocyte cells were infected with rRABV, which is statistically significant compared to mock-infected cells (<1%) (Figure 2B). However, almost 40% of the total splenocytes cells were infected with rRABV-mICAM-1 (Figure 2B). In addition, over 50% of the B cells in culture were infected with rRABV-mICAM-1 compared to about 8% of the B cells infected with rRABV, or 1% background levels in mock-infected cultures (Figure 2C). Of note, the enhanced infection of B cells with rRABV-mICAM-1 was largely due to ICAM-1 incorporation into the virus particle, since pretreatment of sucrose-purified rRABV-mICAM-1 with a neutralizing anti-ICAM-1 antibody significantly reduced infection of B cells in culture (Figure 1D). Together, these data indicate that expression of ICAM-1 from a rRABV-based vaccine enhances infection of primary murine splenocytes, with a propensity for infecting B cells.

**Figure 3:**
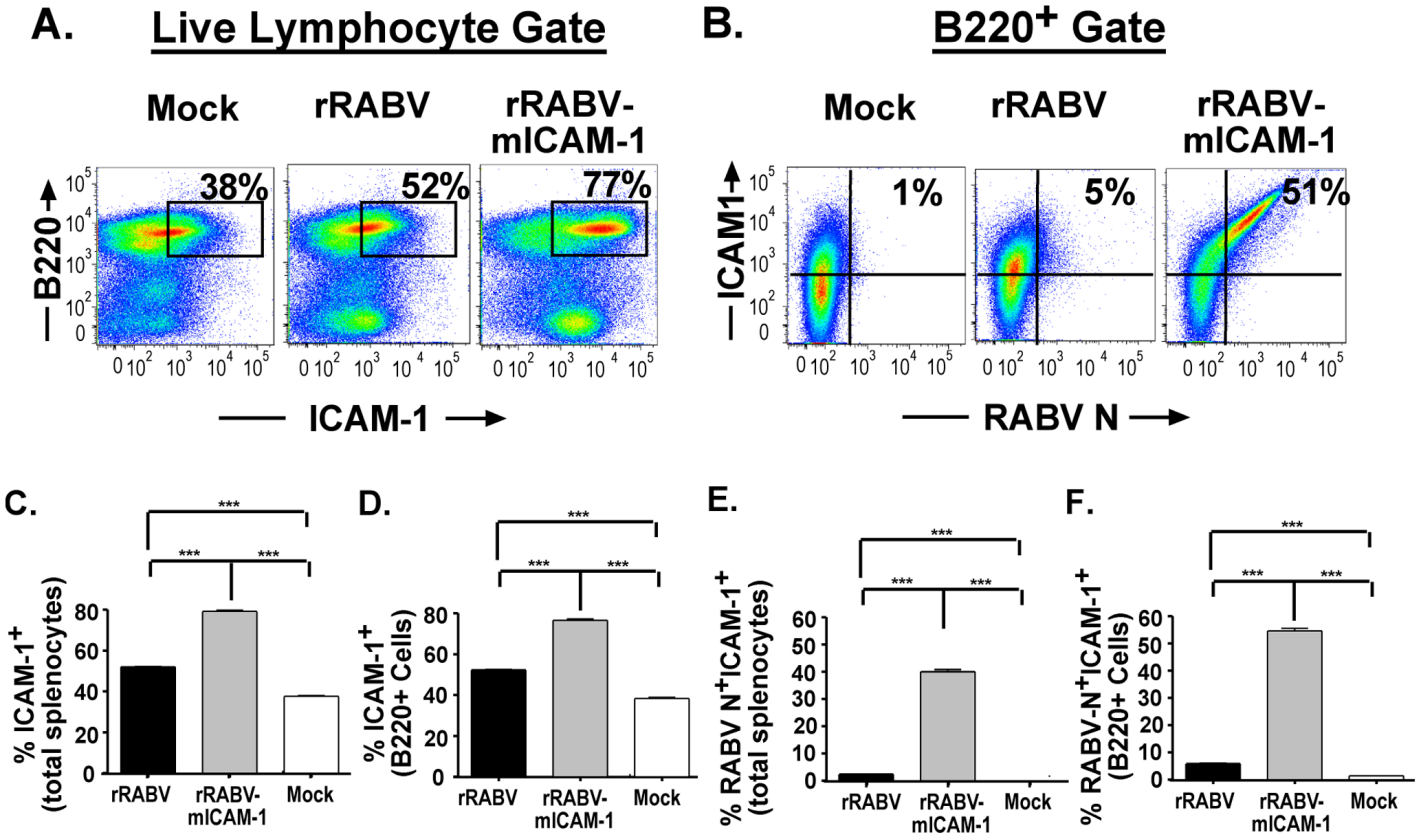
rRABV infection results in the upregulation of ICAM-1 expression, which is augmented by infection with rRABV-mICAM-1. Total naïve primary murine splenocytes were infected as described in Figure 2 and then stained for B220, ICAM-1, and RABV-N for analysis by flow cytometry. **A)** Representative gating strategy of total live lymphocytes gated on ICAM-1 and RABV N staining. **B)** Representative gating strategy of B220^+^ B cells gated on RABV N and ICAM-1. **C)** Percent ICAM-1^+^ cells in the total live lymphocyte population. **D)** Percent ICAM-1^+^ cells in the B220^+^ cell population. **E)** Percent RABV N^+^ ICAM-1^+^ cells in the total live lymphocyte population. **D)** Percent RABV N^+^ ICAM-1^+^ cells in the B220^+^ cell population. To compare two groups of data, we used an unpaired, two-tailed Student's t test. (* p<0.05; **, p<0.01; ***, p<0.001)

Next, we wanted to evaluate the expression levels of ICAM-1 in primary murine splenocytes infected with rRABV and whether expression of ICAM-1 was increased in cultures infected with rRABV-mICAM-1. Figure 3A shows a representative gating strategy of total live splenocytes gated for ICAM-1 and for B220 expression. Figure 3B shows a representative strategy of B220^+^ B cells gated for ICAM-1 and RABV N. Expression of ICAM-1 was significantly increased in total mouse splenocyte cultures (Figure 3C) treated with rRABV compared to mock-infected cultures. This finding indicates that host-cell *Icam1* gene expression is upregulated in murine splenocytes upon infection with attenuated RABV strains. ICAM-1 expression was significantly enhanced in splenocytes cultures treated with rRABV-mICAM-1 compared to rRABV-infected cultures (Figure 3C). Consistent with the pattern of ICAM-1 expression in total splenocytes infected with rRABV or rRABV-mICAM-1, the expression of ICAM-1 within the B cell component of the splenocyte culture was significantly increased in cultures treated with rRABV compared to mock-infected cells, which is statistically increased in B cells infected with rRABV-mICAM-1 (Figure 3D). To confirm that infection enhanced ICAM-1 expression, we analyzed total splenocytes (Figure 3E) or gated B220^+^ B cells (Figure 3F) for infection and ICAM-1 expression (i.e., RABV-N^+^ICAM-1^+^ or B220^+^RABV-N^+^ICAM-1^+^). While rRABV-infected total splenocytes or rRABV-infected B cells upregulated ICAM-1 expression compared to mock-infected cultures, rRABV-mICAM-1-infected cells showed almost 40% of the total splenocytes, or over 50% of the B cells, upregulated ICAM-1 expression. Together, these data indicate that attenuated RABV-based strains induce ICAM-1 expression in primary murine splenocytes, most notably in B220^+^ B cells, which is further enhanced by expressing ICAM-1 from rRABV-mICAM-1.

### rRABV-mICAM-1 Activates Primary Murine B cells *In-vitro* More Effectively Than Does rRABV

**Figure 4:**
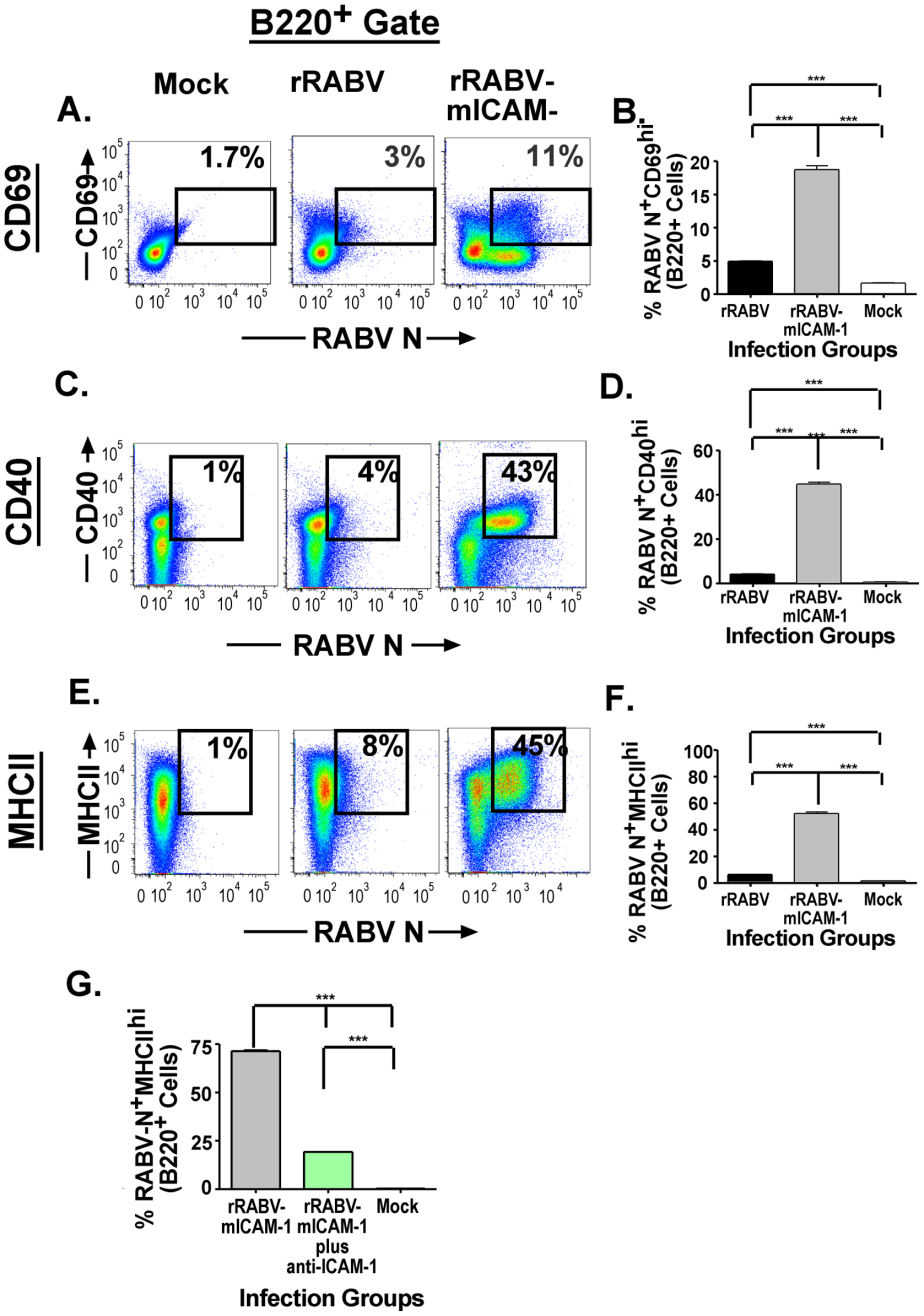
Infection with rRABV results in the upregulation of B cell activation markers, which are enhanced by infection with rRABV-mICAM-1. The infected splenocyte cultures described in Figure 3 were analyzed for the activation of B cells, as measured by the upregulation of cell surface expression of CD69, CD40 or MHCII in RABV N^+^ B cells. Representative gating strategy of B220^+^ B cells from the total live population gated on CD69 (A), CD40 (C) or MHCII (E). The percentage of RABV N^+^CD69^+^ B cells (B), RABV N^+^CD40^+^ B cells (D) and RABV N^+^MHCII^+^ B cells (F) are indicated. **G)** Pretreatment of sucrose-purified rRABV-mICAM-1 with a neutralizing anti-ICAM-1 antibody significantly reduced the expression of MHCII on the infected B cells in culture. To compare two groups of data, we used an unpaired, two-tailed Student's t test. (* p<0.05; **, p<0.01; ***, p<0.001)

Next we wanted to evaluate whether the increased infection noted above in total splenocytes and B cells results in increased activation of primary murine B cells *in-vitro*. Naïve primary murine splenocytes cultures were infected with rRABV or rRABV-mICAM-1, or treated with medium alone (mock-infected). The cells were immunostained two days later for cell-surface expression of the activation markers, CD69, CD40 and MHCII. Figures 4A, 4C and 4E show representative gating strategies of B220^+^ B cells gated for CD69, CD40, or MHCII, respectively, and for RABV N expression. B220^+^ B cells infected with rRABV showed significant upregulation of the activation markers CD69 (Figure 4B), CD40 (Figure 4D) and MHCII (Figure 4F) compared to mock-infected cells. Moreover, the percentage of B220^+^ B cells infected with rRABV-mICAM-1 that express CD69 (Figure 4B, 11%), CD40 (Figure 4D, 43%) and MHCII (Figure 4F, 45%) statistically increased compared to B220^+^ B cells infected with rRABV. Of note, increased B cell activation in response to infection with rRABV-mICAM-1 was largely due to ICAM-1 incorporation into the virus particle, since pretreatment of purified rRABV-mICAM-1 with a neutralizing anti-ICAM-1 antibody significantly reduced MHCII expression in rRABV-mICAM-1-infected B cells (Figure 4G). Together, these data confirm our previous report showing that attenuated RABV-based strains activate naïve primary murine B cells, and that expressing ICAM-1 from rRABV-mICAM-1 enhances B cell activation in RABV-infected cells.

### Kinetics Analyses Shows Enhanced Anti-RABV Antibody Responses in Mice Immunized with a Low Dose of rRABV-mICAM-1 Compared to Mice Immunized with rRABV

**Figure 5:**
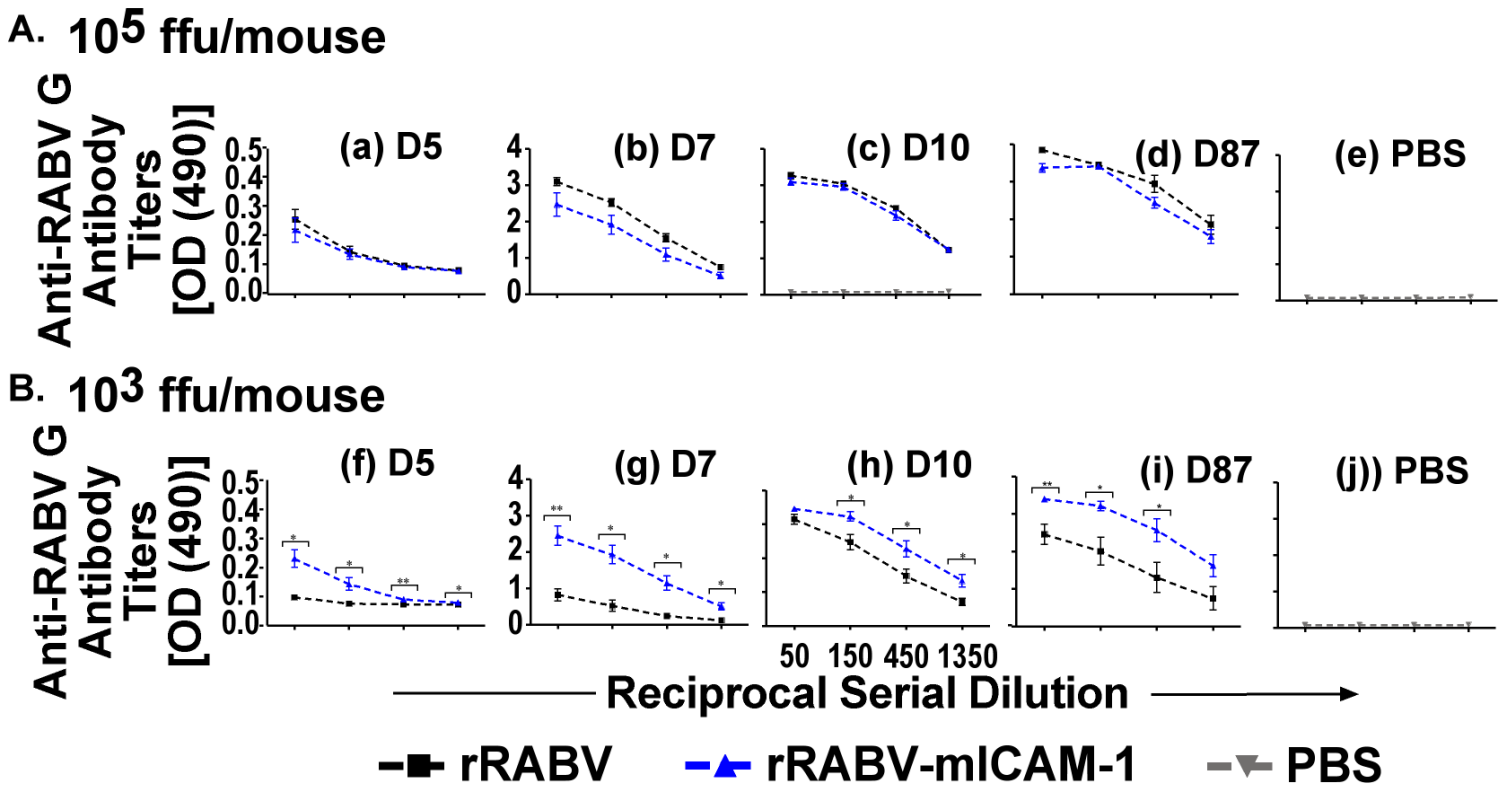
100-fold less rRABV-mICAM-1 is needed to induce comparable immunity in mice compared to mice immunized with 10^5^ ffu/mouse. C57BL/6 mice were immunized i.m. with 10^5^ (A) or 10^5^ (B) ffu/mouse of rRABV and blood collected at the indicated time points. Four serial three-fold dilutions of sera were analyzed by ELISA to determine anti-RABV G antibody titers and presented as OD_490_ of the reciprocal serial dilution. For comparison, sera from PBS-immunized mice (panels e and j) were tested in parallel. Statistical difference in antibody titers by ELISA between two groups of data was determined using an unpaired, two-tailed t test and data is presented at the mean ± SEM. *p<0.05, **p = 0.01−0.001, ***p≤0.001. (N = 5 mice per group). (ffu = focus forming units; OD = optical density)

The above data indicate that rRABV-mICAM-1 infects and activates naïve primary murine B cells more effectively than does rRABV *in-vitro*, suggesting that rRABV-mICAM-1 might promote effective B cell responses *in-vivo*. Furthermore, ICAM-1 binding to LFA-1 on B cells was shown to lower the antigen threshold needed for B cell activation [23], suggesting that a low-dose of a RABV-based vaccine in which ICAM-1 is incorporated into the viral particle might induce potent anti-RABV B cell responses. To investigate anti-RABV antibody responses *in-vivo*, mice were immunized with a high dose (10^5^ ffu/mouse) or a low dose (10^3^ ffu/mouse) of rRABV-mICAM-1 or rRABV. PBS-immunized mice served as negative controls. As shown in Figure 5A, mice immunized with 10^5^ ffu/mouse of rRABV-mICAM-1 showed similar anti-RABV G IgG antibody titers compared mice immunized with the same dose of rRABV at all time points tested (Figure 5A, a–e). However, mice immunized with only 10^3^ ffu/mouse of rRABV-mICAM-1 showed significantly higher anti-RABV G antibody titers as early as five days post-immunization compared to mice similarly immunized with rRABV (Figure 5B, f–j), which were statistically higher at all time points tested thereafter, including 87 days post-immunization, which was the last time point tested. Of note, mice immunized with only 10^3^ ffu/mouse with rRABV-mICAM-1 showed similar antibody kinetics compared to mice immunized with 10^5^ ffu/mouse with rRABV-mICAM-1 or rRABV indicating that 100-fold less ICAM-1-expressing rRABV is needed to induce similar anti-RABV G antibody responses as the parental virus.

### Low-dose rRABV-mICAM-1 Induces VNA Titers *In-vivo* More Rapidly and in Higher Quantities than does rRABV

**Figure 6:**
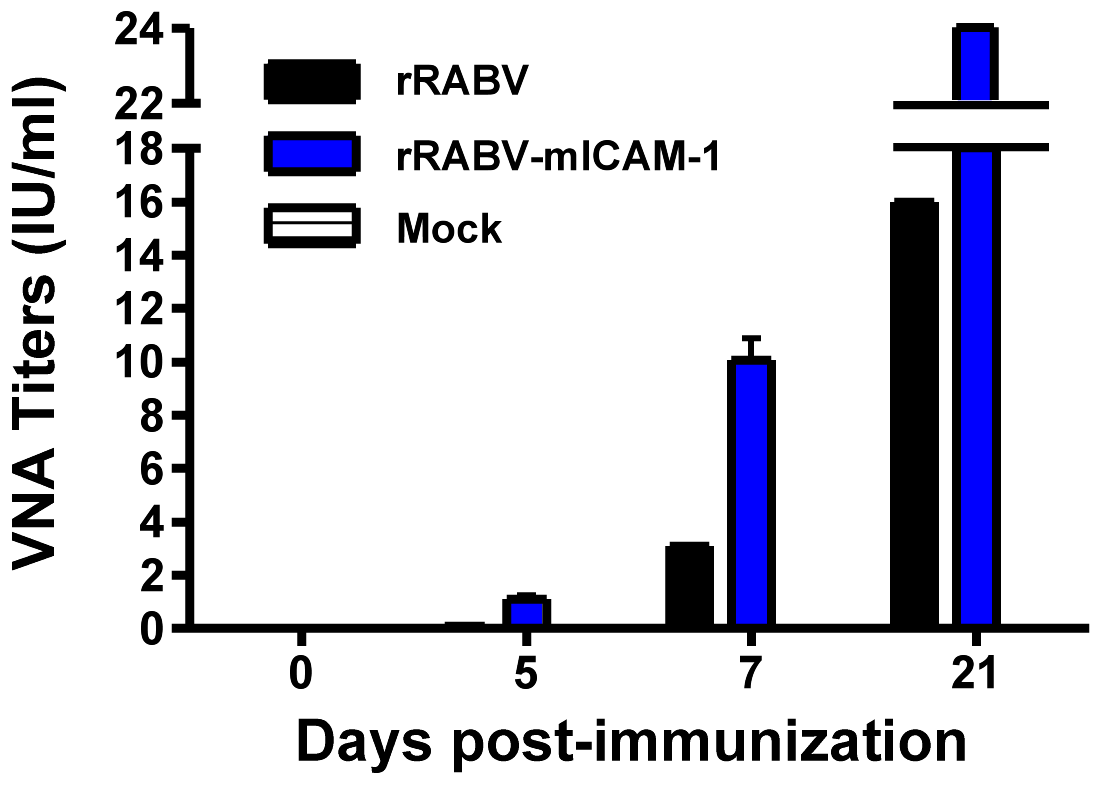
Low-dose rRABV-mICAM-1 induces VNA titers *in-vivo* more rapidly and in higher quantities than does rRABV. Sera from mice immunized with a single dose 10^3^ ffu/mouse with rRABV-mICAM-1 or rRABV were pooled and then VNA titers measured by RFFIT and expressed as International Units (IU/ml). Data represents equal proportions of sera from five mice per group.

VNAs directed against the single viral transmembrane glycoprotein are the primary correlate of protection against RABV infection. The World Health Organization suggests that a VNA titer greater than 0.5 international units (IU)/ml is indicative of a satisfactory immunization [5], [35]–[37]. Consistent with the antibody titers measured by ELISA in Figure 5, VNA titers were similar in mice immunized with 10^5^ ffu/mouse with either rRABV or rRABV-mICAM-1 at all time points tested (data not shown). However, VNA titers in mice immunized with only 10^3^ ffu/mouse of rRABV-mICAM-1 were twice the WHO limit indicative of a satisfactory immunization by five days post-immunization (Figure 6) while less than 0.1 IU/ml were detected in mice immunized with 10^3^ ffu/mouse of rRABV at the same time point. At seven days post-immunization, over 3-fold higher VNA titers were detected in mice immunized with 10^3^ ffu/mouse of rRABV-mICAM-1 compared to rRABV-immunized mice. Higher VNA titers were subsequently detected in rRABV-mICAM-1 immunized mice compared to rRABV-immunized mice at all time points thereafter. VNA titers in mice primed with 10^3^ ffu or 10^5^ ffu of rRABV-mICAM-1 showed similar profiles at all time points measured (data not shown), consistent with serum antibody titers shown in Figure 5.

All together, we show that a recombinant RABV-based vaccine that expresses the murine *Icam1* gene infects and activates naive primary murine B cells more effectively than the parental virus, rRABV. Furthermore, we show that rRABV-mICAM-1 induces significantly higher anti-RABV antibody responses *in-vivo* compared to mice immunized with rRABV alone. VNA titers indicative of a satisfactory immunization were detected as early as five days post-immunization with a single dose of vaccine containing only 10^3^ ffu virus, suggesting expressing ICAM-1 from live RABV-based vaccines may be an excellent single-dose RABV-based vaccine to replace current multi-dose inactivated RABV-based vaccines.

## Discussion

In this study, we demonstrated that a recombinant RABV-based vaccine expressing ICAM-1 (rRABV-mICAM-1), which is incorporated into the virus particle, infects and activates primary naïve murine B cells more effectively than the parental virus, rRABV. Furthermore, we showed that rRABV-mICAM-1 induces significantly higher anti-RABV antibody titers as early as five days post-infection with only a minimum of virus (i.e., 10^3^ ffu/mouse). To the best of our knowledge, this is the first RABV-based vaccine strategy that exploits the immune enhancing properties of ICAM-1.

Interestingly, ICAM-1 is upregulated in total splenocytes, including B cells, infected with rRABV alone, indicating that attenuated RABV-based vaccines promote the expression of cell-derived ICAM-1. The function(s) of cell-derived ICAM-1 expression in response to live RABV vaccination is (are) not known and were not tested directly in this study. For attenuated RABV vaccine strains, the potential exists that the upregulation of ICAM-1 in infected cells influences a wide range of immune functions, including promoting immunological synapses in APC and CD4^+^ T cell interactions (i.e., the immunological synapse) [38], lowering the antigen threshold required for B cell activation thereby reducing the amount of antigen necessary to activate B cells [23], or by promoting T cell activation in B:T interactions [24]. It might be expected that virally encoded ICAM-1 would augment the beneficial effects of ICAM-1 expression on RABV-specific B cell immunity.

The incorporation of ICAM-1 into rRABV-mICAM-1 particles was not expected since the cytoplasmic domain (CD) of the RABV glycoprotein (G) is generally required for incorporation of a foreign protein into the virion particle [39]. Nonetheless, the requirement for the RABV CD appears not to be absolute as shown here and elsewhere [40]. The finding that ICAM-1 is incorporated into the rRABV-mICAM-1 particle may have implications for vaccine design since pre-treating rRABV-mICAM-1 particles with an ICAM-1 neutralizing antibody reduces B cell infection and activation to levels observed with rRABV alone. In addition, ICAM-1 is naturally incorporated into HIV-1 particles, which promotes virus infection by stabilizing virus adhesion to LFA-1 positive cells [21]. The potential exists that ICAM-1 expressed on the surface of the RABV particle enhances and/or prolongs virus:cell interactions, leading to greater virus uptake, infection and activation of B cells that constitutively express LFA-1. In addition, as noted above, ICAM-1 binding to LFA-1 on B cells reduces the antigen threshold needed for B cell activation. Indeed, we observed that rRABV-mICAM-1 induces B cells to produce equivalent antibody titers as 100-fold more of the parental virus, rRABV. Together, we show that attenuated RABV-based vaccines increase ICAM-1 expression in primary murine B cells, which can be augmented by encoding ICAM-1 into the virus genome.

We previously showed that RABV-based vaccines infect primary murine and human B cells, resulting in B cell activation and their ability to prime naïve mouse T cells to proliferate and to secrete IL-2. In the experiments described here, we wanted to determine if increased infection of primary splenocytes or B cells accompanies increased activation in response to infection with rRABV-mICAM-1. Three activation markers were used to study immune cell activation in the context of RABV infection. CD69 is the earliest expressed activation marker on the surface of B and T cells [41], [42] and provides insight into the speed by which rRABV activates primary murine B or T cells. CD40 is a costimulation/survival molecule constitutively expressed on B cells whose expression is upregulated upon B cell:CD4 T cell engagement and is required for optimal B and T cell activation and proliferation [43], [44]. MHCII molecules are loaded with peptide epitopes following internalization and processing of foreign antigen. MHCII-peptide complexes are then expressed on the surface of the B cell for recognition by CD4 T cells specific for the peptide. Engagement of MHCII-peptide complexes with the TCR is the first of two required signals for CD4 T cell activation (reviewed in [45]). Significantly increased expression of CD40 and MHCII on splenic B cells treated with rRABV-mICAM-1 is indicative of an activation state where B cells are capable of presenting antigen to CD4 T cells. In agreement with our previous data [11], we show here that the parental virus, rRABV, increases CD69, CD40 and MHCII expression on the surface of infected B cells. Furthermore, we show that expressing ICAM-1 from the RABV genome significantly increases the expression of these activation markers, suggesting ICAM-1 expression may enhance B cell-intrinsic priming of naive CD4^+^ T cells to proliferate and secrete IL-2 in the context of RABV vaccination. The influence of ICAM-1 expression on the ability of B cells to prime T cells was not directly studied here and remains to be experimentally defined. Nonetheless, the activated B cell phenotype suggests a potential role for virally encoded ICAM-1 to enhance B:T cell interactions.

The primary correlate of vaccine-induced protection against RABV is virus-neutralizing IgG antibodies (VNA). The WHO considers a VNA titer of 0.5 IU/ml to be indicative of a satisfactory immune response to RABV vaccination [5], [35]–[37]. VNA titers in mice primed with only 10^3^ ffu of rRABV-mICAM-1 were 2-fold higher than the presumed level of a satisfactory immunization as early as five days post-prime inoculation, while only background levels of VNA titers were detected in mice immunized with an equivalent amount of rRABV. By day 7 post-immunization, almost a 4-fold increase in VNA titers were detected in mice immunized with 10^3^ ffu/mouse of rRABV-mICAM-1 compared to mice similarly immunized with rRABV. This pattern continued for the duration of the experiment at all time points measured. These data indicate that one low-dose of rRABV-mICAM-1 is highly immunogenic, which is important from a vaccine standpoint. Since similar anti-RABV IgG antibody response were detected using 100-fold less virus (10^3^ ffu vs. 10^5^ ffu), this may allow for reduced manufacturing and preparation costs, helping to reduce the overall cost of RABV PEP.

The proof-of-principle studies described herein provide evidence that RABV-based vaccines can be enhanced by exploiting ICAM-1 expression. Nonetheless, due to safety concerns for the use of a live virus as a vaccine against RABV infection, it is not likely that a live replication-competent vaccine will replace currently used inactivated vaccines. We have previously shown that a replication-deficient RABV-based vaccine in which the matrix (M) gene is deleted (rRABV-ΔM) is both safe and immunogenic in mice and non-human primates [8]. Furthermore, rRABV-ΔM infects and activates primary murine B cells at levels similar as rRABV [11] and induces T cell-independent antibody responses rapidly (neutralizing IgM and IgG) [10]. Based on the preliminary data presented here in the context of replication-competent rRABV-based vaccines, future work will entail testing the potency rRABV-ΔM expressing ICAM-1. Furthermore, while B cells appear to be the major target for rRABV-mICAM-1 infection, rRABV-mICAM-1 appears to also target non-B cells more effectively than rRABV alone. Future work in the context of rRABV-ΔM expressing mICAM-1 will identify a role for these additional cell types in enhanced immunity and animal models of post-exposure protection. Together, we have identified a potential immune enhancing feature that may help increase the utility of highly efficacious and safe RABV-based vaccines, which warrant additional study.
